# Nutritional knowledge, attitude and behaviours regarding Coronavirus Disease 2019 among residents of Gonabad, Iran

**DOI:** 10.1017/S1368980021000033

**Published:** 2021-01-11

**Authors:** Mozhgan Mansoorian, Reza Noori, Shahla Khosravan, Seyed-Amir Tabatabaeizadeh, Nasim Khajavian

**Affiliations:** 1 Faculty of Medicine, Mashhad University of Medical Sciences, Mashhad, Iran; 2 Department of Community Health Nursing & Management Nursing, Faculty of Nursing, Social Development and Health Promotion Research Center, Gonabad University of Medical Sciences, Gonabad 96931-77149, Iran; 3 Department of Community Health Nursing & Management Nursing, School of Nursing, Social Determinants of Health Research Center, Gonabad University of Medical Sciences, Gonabad, Iran; 4 Nutrition and Biochemistry Department, School of Medicine, Social Development and Health Promotion Research Center, Gonabad University of Medical Sciences, Gonabad, Iran; 5 Instructor, Social Development and Health Promotion Research Center, Gonabad University of Medical Sciences, Gonabad, Iran

**Keywords:** COVID-19, Knowledge, Attitude, Behaviour, Nutrition

## Abstract

**Objective:**

Coronavirus Disease 2019 (COVID-19) is a respiratory disease and a major global health-related issue. Knowledge, attitude and behaviours associated with this disease are being developed. Infected animals and consumption of contaminated foods are probably the main sources of this viral infection. Adhering to a healthy diet is effective in preventing patient affliction and recovery. Thus, the present research aims to determine the nutritional knowledge, attitude and behaviours associated with COVID-19 among Residents of Gonabad, Iran.

**Design:**

The present online cross-sectional survey was conducted in March 2020 on 389 people selected through convenient sampling method. The data collection instrument was a questionnaire developed by the present researchers comprising four sections: demographic information, knowledge, attitude and nutritional behaviour. The collected data were analysed statistically in SPSS.

**Setting:**

Gonabad city in Khorasan Razavi in the Northeast of Iran.

**Participants:**

All people at or above 18 years of age.

**Results:**

The present results revealed that the mean age of the participants was 37·3 ± 11·3 years. The mean scores for knowledge, attitude and nutritional behaviour were, respectively, 9·7 ± 1·4, 14·3 ± 3·5 and 29·6 ± 4·2. The mean scores for nutritional knowledge and attitude showed no statistically significant correlation with age, education, occupation, marital status and economic status. However, the mean nutritional behaviour score was significantly higher among women than men (*P* = 0·004).

**Conclusions:**

Participants’ nutritional attitude and behaviour regarding COVID-19, at its early stage in Iran, were average and above average. The public education provided with this regard can have affected this result.

Coronaviruses are in fact a large group of viruses that can affect human and animals. Several types of Coronaviruses have been identified in human that can induce respiratory infections similar to an ordinary cold or a more severe disease such as MERS or Severe Acute Respiratory Syndrome^(1)^ A new type of Coronavirus was found in Wuhan, China in December 2019 which induced Coronavirus Disease 2019 (COVID-19) which is considered as a respiratory disease^(2)^. It soon tuned into a pandemic and spread through many countries including Iran. As reported by the WHO, until 28 May 2020, a total number of 5 593 631 people worldwide were afflicted with the disease and the mortality rate was 353 334. On this date, the affliction rate in Iran was 141 591 and the mortality rate was 7546^(3)^. Though the commonest symptoms of the disease are fever and respiratory problem, about one-sixth of the exposed may experience severe respiratory problems^(1)^. Yet, the gastrointestinal symptoms such as diarrhoea, vomiting and abdominal pain have been reported among patients. Rectal swab samples showed that when the respiratory symptoms are gone, the virus can be separated from the faeces^(4)^. Although the main way of transmitting this disease among humans is through direct or indirect contact with infectious droplets^(5)^, there is a high probability of oral/faecal transmission of the virus especially through faecal aerosol possibly produced in toilet. This can help to manage, control and prevent the disease^(4)^. Nutrition is always a health-related concern. The prevalence of the disease among obese and diabetic patients shows the effect of nutrition on one’s exposure to the disease and the long-lasting adverse effects of COVID-19^(6)^. As there is yet no definite treatment or vaccine for the disease, the best preventive measures are the regular washing of hands, wearing masks using complementary medicine and improving the immune system. Inadequate knowledge of the disease and fear of its consequences created certain nutritional beliefs and behaviours such as alcohol consumption, unapproved by WHO, to prevent the disease among people^(7)^. WHO (2000) drew attention to the effect of commonplace nutritional beliefs on the disease epidemic^(8)^. There is not definite treatment or vaccine available yet. However, nutrition behaviour is related to the immune system. Thus, the present research aimed to determine the nutritional knowledge, attitude and behaviours associated with COVID-19 among Residents of Gonabad, Iran.

## Methods

### Setting

The present cross-sectional online survey was conducted in 2020, one month after the COVID-19 epidemic occurred in Iran. The research population comprised urban and rural residents of Gonabad in Khorasan Razavi, in the Northeast of Iran. The inclusion criteria were willingness to participate in research, the minimum age of 18 years, no mental disability and ability to answer the online questionnaire survey. The exclusion criterion was not completing the questionnaire survey within the required time. The sample size was estimated based on Morgan and Krejcie’s Table, and the probability of attrition in the sample was estimated at 400. Once the research project was approved by the deputy of research and technology in Gonabad University of medical sciences, all required permissions were gained and the sampling was done through convenient method. To adhere to the home quarantine requirement and prevent the disease, if an individual consented to participate in the research and met the inclusion criteria, s/he has sent the questionnaire via email or social networks such as WhatsApp or Telegram.

### Data collection

The data collection instrument in the present research was a questionnaire with four sections, the first of which addressed participants’ demographic information such as gender, age, marital status, education and occupation. The second section included thirteen items about the nutritional knowledge of COVID-19 such as ‘Can contaminated dishes transmit COVID-19?’ The response was either yes or no, and the correct answer was scored 1 and incorrect answer 0. The third section explored participants’ nutritional attitude towards COVID-19 and consisted of thirteen items to be rated on a Likert scale: disagree, no opinion and agree. Each item was scored between 0 and 2. The last section of the questionnaire included twenty items that dealt with nutritional behaviour regarding COVID-19 and involved such items as: ‘Before eating or drinking anything, I wash my hands’. The items were to be answered on a Likert scale: always, sometimes and never. Each item was scored between 0 and 2. The questionnaire content was based on WHO official website, and we asked the participants who reply to questions with their prior knowledge (do not use Google or other information sources). The validity of the questionnaire was confirmed by a panel of experts in nutrition, health and nursing domains. Yet, for the critical conditions, the reliability of the questionnaire was not tested.

### Statistical analysis

The data were analysed via SPSS-16. For data description, percentage, frequency, central tendency and distribution indices such as mean and sd were used and, for inferential statistics, tests such as Kolmogorov–Smirnov test (to test normal distribution), *t* test, ANOVA, Pearson’s Correlation Coefficient were run. The significance level was set at 0·05.

## Results

From among the 400 questionnaires returned, eleven were incomplete and thus discarded. Eventually, 389 questionnaires were analysed. The mean age of participants was 37·3 ± 11·3 years. Among the participants, 340 (87·4 %) stated that they had no special disease such as diabetes, hypertension, CVD or respiratory diseases. Moreover, 388 (99·7 %) participants acknowledged that they had never been hospitalised before for any symptom of Coronavirus disease. Table 1 includes the distribution of other demographic features as indicated in the present research.

**Table 1:** Demographic information

Variable	Level	Frequency	%
Gender	Male	205	52·7
	Female	184	47·3
Marital status	Married	340	87·3
	Single	43	11·1
	Divorced	5	1·3
	Widowed	1	.3
Economic status	High	6	1·5
	Low	59	15·2
	Average	324	83·3
Education	Literate (able to read and write)	14	3·6
	Below diploma	44	11·3
	Diploma	107	27·5
	Associate degree or bachelor’s degree	168	43·2
	Master’s degree or higher	56	14·4
Occupation	Retired	27	6·9
	Unemployed	20	5·1
	Housewife	108	27·8
	Student	9	2·3
	University student	20	5·1
	Freelance	75	19·3
	Employed	94	24·2
	Other	36	9·3

Table 2 indicates the mean scores for nutritional knowledge, attitude and behaviour regarding COVID-19. As the results revealed, the participants’ mean scores for nutritional knowledge and behaviour were above average and the mean score for nutritional attitude was about the average.

**Table 2:** Participants’ mean scores for nutritional knowledge, attitude and behaviour

Variable	Mean	sd	Minimum	Maximum
Nutritional knowledge	9·7	1·4	5	13
Nutritional attitude	14·3	3·5	5	24
Nutritional behaviour	4·2	29·6	15	40

Concerning the items within the knowledge section, Table 3 shows that 88·3 % of the participants often perceived food as a way of transmitting the disease. Besides, 82 % knew that drinking a lot and eating healthy cooked food were effective in preventing the disease. 99·1 % knew that eating foods rich in vitamins D, C, B, S and E (contrary to taking supplementary food) strengthened the immune system. Sixty-nine percentage knew that eating proteins and 54·8 % knew that taking Fe, Zn and Se helped to strengthen the immune system and prevent the disease.

**Table 3:** Distribution of participants’ answers to the knowledge section of questionnaire

#	Item	Yes (%)	No (%)
1	Can eating contaminated food transmit COVID-19?	88·3	11·7
2	Can drinking liquids adequately help to maintain body immunity?	91·8	8·2
3	Does consuming certain foods affect the prevention and treatment of COVID-19?	88	12
4	Does consuming food materials rich in vitamins D, C, B, A and E strengthen the immune system?	99·1	0·9
5	Does a high dose of multivitamins help to a rapid treatment of COVID-19?	16	84
6	Does consuming greasy and salty food weaken the immune system?	81·5	18·5
7	Does consuming carbohydrates help to produce energy the body needs during the disease?	61·5	38·3
8	Do food materials rich in protein strengthen respiratory muscles during COVID-19?	69	31
9	Should we consume more plant oil than animal oil during COVID-19?	55	45
10	Does consuming Fe, Zn and Se help to strengthen body immunity?	92	8
11	Does an overconsumption of such materials as ginger and garlic affect body metabolism, absorption and effect of drugs?	54·8	45·2
12	Can contaminated dishes transmit COVID-19?	96·8	3·2
13	Should we cook food materials to kill Coronavirus?	82	18

COVID-19, Coronavirus Disease 2019.

Table 4 shows the participants’ attitudes and beliefs. The participants believed that eating forbidden foods in Islam (32 %), eating the meat of wild animals (42 %) and consuming fast food (59·2 %) were the main factor involved in COVID-19 and that consuming fruits and vegetables (93 %) and a diverse diet (42 %) along with supplementary foods (57·7 %) were successful in preventing the disease.

**Table 4:** Distribution of participants’ answers to the attitude section of questionnaire

#	Item	Agree	No opinion	Disagree
1	I believe that consuming foods forbidden in Islam is the main cause of COVID-19.	32	43	25
2	A vegetarian diet is better than other diets.	26·5	28·3	45·2
3	Consuming fresh (uncooked) foods is of a higher nutritional value.	17	22	61
4	A diet has no effect on the emergence of a viral disease.	25·8	27·6	46·6
5	Eating the meat of wild animals can cause the disease.	42	38	15
6	Consuming fruits and vegetables is effective in preventing and treating the disease.	93	4·7	2·3
7	A diet marked by variety and balance can prevent the disease.	42	21	37
8	Consuming fast food is the main cause of the disease.	59·2	20·4	20·4
9	Supplementary foods should be included within our diet.	57·7	23·3	18
10	Paying attention to the heath of food materials can prevent the disease.	53	19	28
11	Islamic medical advice can help to prevent and treat the disease.	48·7	31·8	19·5
12	I think herbal medicine can help to treat the disease.	37·6	30	32·4
13	An overconsumption of hot-tempered foods such as garlic and ginger can prevent the disease.	44·5	24·7	30·8

Concerning the behaviour section of the questionnaire, Table 5 shows that purchasing food materials at healthy places (Health-certified food preparation and distribution centres that follow personal hygiene and the environment) (88 %), washing hands before eating/drinking (84·3 %), using personal dishes to eat (72·2 %), washing and sanitising fruits and vegetables (80·4 %) and avoiding crowded and unhealthy places (non-certified food preparation and distribution centres that do not follow personal and environmental hygiene) (79 %) were among the main behaviours participants always did to prevent the disease.

**Table 5:** Distribution of participants’ answers to the behaviour section of questionnaire

#	Item	Always	Sometimes	Never
1	I wash my hands before eating or drinking anything.	84·3	15·7	0
2	I avoid eating fresh (uncooked) food.	76·7	20·7	2·6
3	Before consuming fruits and vegetables, I sanitise them.	80·4	18·2	1·5
4	I avoid eating at crowded and unhealthy places.	79	19	2
5	I avoid eating fast foods.	57·6	38·4	4
6	I use more packed foods.	33·2	54·8	12
7	I use supplementary foods more to strengthen my immune system.	12·5	59·6	27·9
8	I consume hot-tempered food materials such as ginger, cinnamon and saffron to prevent COVID-19.	37·8	59·3	2·9
9	To further strengthen my immune system, I consume fruits containing vitamin C such as orange, kiwi and tangerine.	64·4	34·4	1·2
10	To prevent the disease, I consume more vegetables, salad, garlic and onion.	43	54·7	2·3
11	To strengthen the immune system, I consume dairy products rich in vitamin D and sea food.	20	66·4	13·6
12	To prevent infectious diseases, I tend to consume hot drinks.	43	51	6
13	If affected, I tend to consume food materials rich in protein such as eggs, meat and cereals.	45·3	52	2·7
14	I use rich sources of minerals more such as Se and sea food.	8·7	70·9	20·3
15	To prevent the disease or while afflicted, I drink 6–8 glasses of water or other liquids every day.	53·2	43	3·8
16	When afflicted with respiratory infections, I use more carbohydrates such as rice, bread types and macaroni.	20·6	62·4	17·1
17	I purchase food materials at healthy places.	88	11·7	0·3
18	I use personal dishes while eating.	72·2	22·8	5
19	When afflicted with respiratory infections, I tend to have more meals but of a smaller size.	42·5	49·6	7·9
20	I eat the meat of wild animals.	0·9	4·1	95

Table 6 indicates the correlation between participants’ nutritional knowledge, attitude and behaviour and their demographic information. As it can be observed, women had a better nutritional perceived behaviour than men (*P* = 0·004). Moreover, the mean score for behaviour diverged across marital statuses (*P* = 0·001). *Post hoc* test revealed the difference between single and married levels. Overall, participants of a higher age showed better nutritional perceived behaviour than others (*P* = 0·027).

**Table 6:** Correlation between nutritional knowledge, attitude and behaviour and several demographic features

Variable		Nutritional knowledge		Nutritional attitude		Nutritional behaviour	
		Mean	sd	Mean	sd	Mean	sd
Gender	Male	9·8	1·4	14·3	3·6	29·1	4·2
	Female	9·7	1·5	14·4	3·5	30·3	4
	* *P*-value	0·856		0·940		0·004	
Marital status	Married	9·7	1·4	14·3	3·5	29·9	4
	Single	9·8	1·5	14·1	3·6	27·3	4·9
	Widowed	11	0	17	0	34	0
	Divorced	11·2	1	15·4	3·1	32	1·2
	† *P*-value	0·132		0·782		0·001	
Economic status	High	10	1·4	15·8	0·7	29	4·8
	Average	9·8	1·4	14·2	3·5	29·9	3·9
	Low	9·5	1·5	14·8	3·5	28·3	5·1
	† *P*-value	0·485		0·323		0·026	
Education	Literate (able to read and write)	10·1	1·1	14·5	4·8	30·7	3·6
	Below diploma	9·4	1·5	14·8	3·9	29·8	4·2
	Diploma	9·6	1·4	14·8	3·4	29·5	4·4
	Associate degree or Bachelor’s degree	9·9	1·4	14	3·3	29·5	4·3
	Master’s degree or higher	10	1·3	14·2	3·6	30·1	3·7
	† *P*-value	0·693		0·095		0·392	
Occupation	Retired	10	1·1	14·5	3·4	30·4	2·8
	Unemployed	9·8	1·1	16·4	3·7	28	5·5
	Housewife	9·8	1·5	14·4	3·5	30·6	3·7
	Student	9·2	1·7	14·3	2·5	28·2	2·7
	University student	10	1·2	13·6	3	26	5
	Other	9·2	1·4	14·5	3·5	29·5	3·9
	Freelance	9·6	1·4	14·5	3·5	29·5	3·9
	Employee	9·7	1·3	13·9	3·7	30·3	3·4
	† *P*-value	0·173		0·347		0·001	

* Independent-sample *t* test.

† ANOVA test.

No statistically significant difference was found between economic status and the mean scores for nutritional knowledge and attitude. Yet, the mean score for behaviour did not diverge across different economic statuses (*P* = 0·026). The *post hoc* test revealed different nutritional behaviour between the two levels of economic status, average and low levels and also revealed differences between university students and employees on the one hand and university students and housewives on the other.

## Discussion

The present research aimed to explore the nutritional knowledge, attitude and behaviour associated with COVID-19. The overall results revealed that the majority of participants were male, married, holding a diploma or higher degree, average economic status and employed.

The mean score for the participants’ nutritional knowledge was above the average. Though there have been several studies conducted in other countries such as China^(2)^, Thailand^(9)^, USA^(10)^ and Jordan^(11)^, no similar research was found with a focus on nutrition regarding COVID-19 at the time of the present research to compare the results with. Regarding knowledge, 88·3 % of the participants perceived food as a way of transmitting the disease. In some other work of research in Singapore, similarly, the participants often mentioned food as one way of transmitting the disease^(12)^. In two studies conducted in China and USA, respectively, 91 % and 54 % of the participants answered correctly to the item: ‘Eating the mean of wild animals can cause the disease’^(2,10)^. This item, in the present research, belonged to the attitude section and 42 % of the participants agreed while the rest either disagreed or had no opinion. There was a significant difference between the percentages of correct answers Chinese participants gave and that of US and Iranian, which is probably explained by the different diets common in these countries. The origin of this viral disease, epidemiologically speaking, was primarily the whole sale of sea food in China and wild animals were the main source of the disease^(13)^. Infected animals and consuming contaminated food are probably the main source of viral infections^(8)^. Besides implementing the quarantine, closing animal markets in China was a key measure taken in China to prevent the epidemic of the virus. Purchasing healthy food materials and changing the traditional habit of eating wild animals (and their products) have been recommended as a preventive behaviour^(5,13)^.

Concerning the other knowledge-related items associated with COVID-19, the majority of answers provided corresponded to the existing knowledge at the time of the research and the recommendations by WHO and educational programmes broadcast on Iran TV. Inadequate food materials and vitamins such as A, C and D and weakened immune system would raise the chances of the disease^(14,15)^. Consuming vegetables and salad and fruits containing vitamin C, carrot, pumpkin and onion in the main meals, proteins such as egg and avoiding fast foods, raw or medium-cooked food, avoiding eating at unhealthy places and washing fruits and vegetables are commonly recommended to better prevent the disease^(15,16)^. Moreover, avoiding high doses of vitamin and mineral supplementary and overconsumption of such food materials as ginger or garlic are recommended that can affect metabolism and disrupt absorption and effect of drugs^(17,18)^. Eating fresh food, drinking enough water and liquid, balanced consumption of oil and less salt and sugar, avoiding eating at crowded places outside home and consultation in the case of affliction with the disease are among the recommendations made by WHO for adult nutrition in COVID-19 crisis^(19)^ that majority of participants answered correctly.

Concerning attitude, the majority of participants disagreed with these statement that eating the meat of wild animals forbidden in Islam led to the disease. Though this item was not asked from the participants, as COVID-19 is introduced as a viral respiratory disease, there are chances that the participants considered this disease primarily viral. Similarly, Butler and Barrientos in their research drew attention to the prevalence of the disease among diabetic and obese patients. This shows that the eating style especially Western eating style which involves saturated oil, high sugar and low fibre can weaken the immune system and reduce B and T lymphocytes. However, healthy fresh food with its anti-inflammatory effect even in the obese can help to prevent the disease and improve their conditions. Therefore, a healthy life style especially one marked by consuming more fibre and unsaturated oil and antioxidants and refraining from foods containing high levels of sugar and unsaturated oil is recommended to strengthen the immune system^(6)^. Zhang and Liu, in their research, recommended the use of foods rich in vitamins A, B, C, D and E as a public intervention to strengthen the general system to prevent and treat the disease^(15)^. Some other research in Nigeria showed that about 11 % of the participants perceived consuming garlic, ginger and certain herbs and soups as effective ways of preventing the disease^(20)^.

As for nutritional behaviours associated with COVID-19, participants showed that they often abided by the recommendations made by WHO broadcast in national TV. These included washing hands, cooking foods, washing and consuming fruits and vegetables and avoiding eating out in crowded places, consuming prepacked foods, consuming water, garlic and ginger. As study conducted in Jordan showed that university students ate garlic, ginger syrup and honey to prevent the disease. The results revealed no statistically significant difference between medical and non-medical university students^(11)^. Similarly, in the present research, no statistically significant divergence was found in terms of knowledge and attitude between university students and others. However, women and more specifically housewives showed healthier nutritional behaviour based on updated knowledge of nutrition at the time of COVID-19 than others. As women have long played a key role in promoting and supporting food consumption in family, they also play a key role through cooking healthy food to promote healthy behaviours in family. Thus, this result is desirable considering the probable effect of healthy nutrition on preventing COVID-19. Knowledge and level of education on the one hand and behaviour and economic status on the other showed to be correlated. A similar correlation was found in the study in American context among the black who were also less educated and had also lower income^(10)^.

### Strengths and limitations

The present study has the first study to assessment nutritional knowledge, attitude and behaviours regarding COVID-19 among residents of Iran. One limitation of the present research was the deficient and incomplete knowledge of COVID-19 at the time of conduction. Moreover, there were chances that the participants answered items they did not understand correctly. Considering the cross-sectional nature of the research and that the majority of participants were of the average economic status, the results could not be adequately in similar generalised to other groups and geographies. It seems that further similar research is required in different populations and groups of different education levels to increase understanding of nutritional knowledge, attitude and behaviour regrading COVID-19.

## Conclusion

The present results showed that the nutritional knowledge, attitude and behaviours of Gonabad residents regarding COVID-19 has been appropriate. Thus, it can be concluded that the relevant national educational programmes within the limited time have managed to correct the nutritional knowledge, attitude and behaviour of people in Gonabad (a city marked by a high level of infection). Yet, continuing the health education programme for the public with a focus on nutrition needs to be corrected and increased.
